# A Comparative Study on Feature Extraction Techniques for the Discrimination of Frontotemporal Dementia and Alzheimer’s Disease with Electroencephalography in Resting-State Adults

**DOI:** 10.3390/brainsci14040335

**Published:** 2024-03-29

**Authors:** Utkarsh Lal, Arjun Vinayak Chikkankod, Luca Longo

**Affiliations:** 1 Department of Computer Science and Engineering, Manipal Institute of Technology, Manipal 576104, Karnataka, India; utkarsh.lal@learner.manipal.edu; 2 Department of Information and Communication Technology, Manipal Institute of Technology, Manipal Academy of Higher Education, Manipal 576104, Karnataka, India; arjun.cv@manipal.edu; 3 Artificial Intelligence and Cognitive Load Lab, the Applied Intelligence Research Centre, Technological University Dublin, D07 H6K8 Dublin, Ireland

**Keywords:** electroencephalography, neural signal processing, feature extraction techniques, supervised learning

## Abstract

Early-stage Alzheimer’s disease (AD) and frontotemporal dementia (FTD) share similar symptoms, complicating their diagnosis and the development of specific treatment strategies. Our study evaluated multiple feature extraction techniques for identifying AD and FTD biomarkers from electroencephalographic (EEG) signals. We developed an optimised machine learning architecture that integrates sliding windowing, feature extraction, and supervised learning to distinguish between AD and FTD patients, as well as from healthy controls (HCs). Our model, with a 90% overlap for sliding windowing, SVD entropy for feature extraction, and K-Nearest Neighbors (KNN) for supervised learning, achieved a mean F1-score and accuracy of 93% and 91%, 92.5% and 93%, and 91.5% and 91% for discriminating AD and HC, FTD and HC, and AD and FTD, respectively. The feature importance array, an explainable AI feature, highlighted the brain lobes that contributed to identifying and distinguishing AD and FTD biomarkers. This research introduces a novel framework for detecting and discriminating AD and FTD using EEG signals, addressing the need for accurate early-stage diagnostics. Furthermore, a comparative evaluation of sliding windowing, multiple feature extraction, and machine learning methods on AD/FTD detection and discrimination is documented.

## 1. Introduction

Alzheimer’s disease (AD) systematically destroys brain neurons over time [1]. This neurodegenerative disorder progressively leads to cognitive decline, notably in brain regions associated with memory. AD arises from various factors, including environmental influences, vascular diseases, head injuries, genetic predispositions, and, particularly, ageing. More than 50 million people are diagnosed with AD around the globe [2], with this type of disorder significantly contributing to elderly disability and dependency and defining the seventh most crucial cause of death. Similarly, frontotemporal dementia (FTD) is a neurodegenerative disorder that leads to issues associated with communication challenges and behavioural changes. Diagnosing these disorders progresses through several stages: an asymptomatic early pre-clinical phase, a period of mild cognitive impairment, and finally, dementia [3]. As a consequence, diagnoses at an early stage are crucial. A diagnosis can be achieved by utilising physical exams, cerebrospinal fluid tests, cognitive and language tests, and urine and blood tests. Brain scans can also be adopted, such as Computed or Positron Emission Tomographies (CT/PET) and Magnetic Resonance Imaging (MRI) techniques [3]. While brain scans offer detailed spatial resolution, they lack the temporal precision to capture dementia’s evolving symptoms. Electroencephalography (EEG), with its superior temporal resolution, can detect subtle brain activities essential for understanding the dynamic neural interactions in dementia. Moreover, EEG’s cost-effectiveness, accessibility, and ability to provide real-time brain activity monitoring make it suitable for broad screening and diagnostics. However, the volume of recorded EEG data and its inherent artefacts often pose difficulty in identifying critical biomarkers and, thus, accurately diagnosing neurodegenerative disorders. Different research approaches have appeared over the years to mitigate these technical issues. Moreover, there is an abundance of EEG denoising pipelines present in the literature, as previous studies have applied various techniques for extracting high-level features from EEG data, such as wavelet transforms [4], fractal dimensions [5], entropy-based features [6], and the Hurst exponent [7]. Similar techniques have been used for extracting features for detecting and diagnosing Parkinson’s [8] epilepsy [9,10], schizophrenia [11], and other neurological disorders. However, there is limited research on comparing and assessing the utility of such feature extraction techniques for discriminating between Alzheimer’s and frontotemporal patients, as well as from healthy controls (HCs).

This study’s objectives are as follows:

1. To evaluate the effectiveness of sliding windowing, feature extraction techniques, machine learning models, and their pipelines to detect and discriminate AD, FTD, and HC biomarkers.
2. To identify brain regions affected by AD and FTD and verify if these regions align with standard medical tests.

Our Research Question (RQ):

RQ: *How does the choice of sliding windowing, feature extraction measures, and machine learning models affect the detection and differentiation of AD and FTD biomarkers in EEG data?*

We examined 50% and 90% overlaps for sliding windowing, multiple feature extraction techniques—Higuchi Fractal Dimension, Singular Value Decomposition (SVD) Entropy, Zero Crossing Rate, Detrended Fluctuation Analysis, and Hjorth parameters—to extract salient high-level features from EEG signals and supervised machine learning techniques—K-Nearest Neighbors (KNN), Random Forest (RF), XGBoost, and Extra Trees (ET)—to discriminate frontotemporal dementia, Alzheimer’s disease, and control groups.

The remainder of this paper includes a description of related work for AD and FTD detection (Section 2), and the introduction of a comparative design study for feature extraction from EEG data employing machine learning classification techniques (Section 3). Findings are subsequently presented and critically discussed in Section 4. The contribution to the body of knowledge is explicated in Section 5 by synthesising this research and delineating future avenues of work.

## 2. Related Work

Several investigations have been conducted for diagnosing Alzheimer’s disorder from biomarkers extracted from electroencephalographic data. Few researchers have employed Hjorth parameters, which are specific statistical properties of EEG data [12]. Others have employed entropy-based features [13], standard measures adopted within biomedicine that represent the degree of disorder of an EEG signal. Various research studies have been based upon the computation of EEG source localisations and the extraction of connectivity features of the cortical region [14] for identifying AD-induced brain network disruptions. Various feature extraction techniques from EEG data exist, and the resulting high-level features are often aggregated and analysed using machine learning. A recent study focused on classifying EEG data from a large dataset of 890 subjects across three categories: healthy controls, mild cognitive impairment subjects, and patients with Alzheimer’s [15]. Another study scrutinised standard EEG pre-processing techniques using exploratory analysis and highlighted their importance in identifying early AD indicators [16]. Further research has also emphasised meticulous pre-processing and feature extraction techniques, employing methods like Kolmogorov Complexity [17], Discrete Wavelet Transform [18], and Spectral Entropy.

Parallel efforts in FTD diagnosis are also noteworthy. A study presented an automatic FTD detection technique by employing Independent Component Analysis (ICA) in the pre-processing phase of the EEG data and a Light Gradient Boosting (LGB) for classification, achieving an 80.67% accuracy [19]. Another study emphasised the discovery of crucial biomarkers in differentiating FTD from other neurodegenerative diseases, focusing on serum and cerebrospinal fluid markers [20]. Similarly, various other forms of dementia, such as the vascular one and mild cognitive impairment, were contrasted using EEG data [21]. This approach combined Wavelet Transform in the EEG pre-processing phase jointly with Independent Component Analysis (ICA). Feature extraction techniques, including Spectral, Permutation, and Tsallis Entropy, were used to augment original EEG data. The study employed machine learning to train supervised models using Support Vector Machines and the K-Nearest Neighbours learning algorithms, coupled with neighbourhood-preserving QR-decomposition for dimensionality reduction based on fuzzy logic. Similarly, researchers performed feature selection via the improved binary gravitation search approach, leading to a higher classification accuracy of patient groups [22].

Machine and deep learning-based applications have been widely adopted for solving supervised AD detection with EEG data analysis [18,23,24,25]. For example, Convolutional Neural Networks (CNNs) have been trained on functional brain connectivity features to detect AD and other neurological disorders automatically [26]. Similarly, a feed-forward neural network was trained on DNA methylation and gene expressions after employing dimensionality reduction techniques. Another study used convolutional auto-encoders to classify AD, mild cognitive impairment, and healthy control subjects utilising time–frequency high-level features generated from the application of Continuous Wavelet Transform [27].

Although deep learning has demonstrated a superior capacity to develop models for automatically learning and integrating salient features from EEG data for an improved classification accuracy [28], they are often considered difficult to interpret and explain. Studies employing more straightforward learning methods, such as logistic regression, have shown that optimally pre-processed data can still lead to a higher model’s performance, and complex learning strategies are not often helpful [29]. This suggests that external but transparent and interpretable techniques can often help extract salient features from EEG data better than automated deep learning methods to classify neurodegenerative disorders. Along with the use of more transparent methods, a novel study focused on multimodal EEG and cerebrospinal fluid-related data to distinguish early-onset Alzheimer from FTD subjects by adopting microstates theory and spectral analyses [30]. In detail, EEG microstates are short time intervals of stable scalp potential fields. This study demonstrates how abnormalities associated with early-onset AD subjects could be detected by analysing the variation in EEG microstate duration and global field power peaks correlating with clinical severity and cerebrospinal fluid biomarkers. Another study extracted statistical features from EEG frequency bands trained with Decision Trees and Random Forests to discriminate Alzheimer’s and frontotemporal dementia subjects. These algorithms are not only more straightforward and interpretable than deep learning algorithms, but they lead to the development of models with remarkable accuracy [31].

Besides the transparency offered by simpler machine learning algorithms or the capacity of deep learning to deliver highly accurate predictive models, there is the technical problem of extracting salient features from large datasets with a reasonable computational complexity in computer memory and time [28,32,33]. Consequently, external techniques for extracting meaningful high-level features from EEG data are not only helpful for transparency and interpretability but are often required for dimensionality reduction and, thus, for a significant decrement in the computational resources required to train predictive models. In this direction, often, Fast Fourier Transforms [34], and Discrete Wavelet Transforms [35] have proven helpful in extracting features from EEG data before training models with machine learning algorithms. Similarly, Multiway Array Decomposition [36], Principal Dynamic Mode (PDM) analysis [37], Singular Value Decomposition [9], and Principal Component Analysis [38] have all demonstrated utility in such endeavour.

In summary, the body of research on identifying Alzheimer’s disorders and frontotemporal dementia using electroencephalographic data is extensive, along with the adoption of machine and deep learning to develop improved predictive models. However, the problem of evaluating the utility of various ad hoc interpretable techniques to learn salient features and biomarkers from EEG signals associated with Alzheimer’s disorders and frontotemporal dementia, often used as inputs to the aforementioned learning techniques, is elusive.

## 3. Materials and Methods

**Figure 1:**
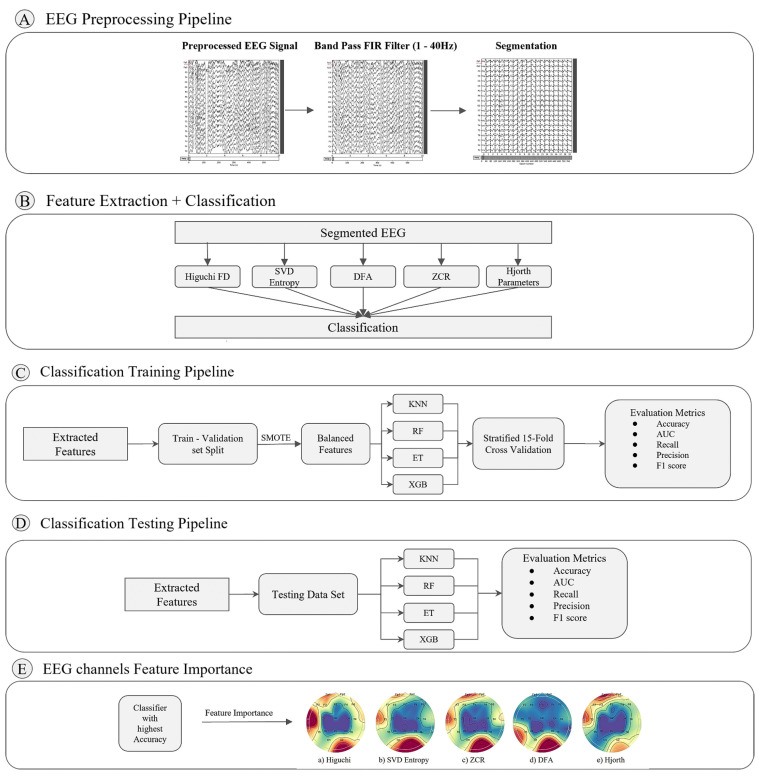
A pipeline for the discrimination of subjects having frontotemporal dementia and Alzheimer’s disorder.

This section introduces an empirical work that compares various feature extraction techniques from EEG data for discriminating subjects with Alzheimer’s disorder and frontotemporal dementia from healthy adults. Figure 1 shows a synthesis of such a novel pipeline that is divided into five phases: (A) a pre-processing pipeline to denoise EEG data and to segment it with a sliding window technique; (B) a phase where various feature extraction techniques for EEG data are contrasted, along with straightforward supervised learning algorithms for classifying frontotemporal dementia subjects from those having Alzheimer’s disorder; (C) a training phase for automatically aggregating the extracted features from the previous step towards predictive models; (D) an evaluation of such models with unseen testing data across various evaluation metrics; (E) an analytical phase for establishing the importance of EEG channels in the discrimination of Alzheimer’s and frontotemporal dementia subjects.

### 3.1. Dataset

A dataset published in the OpenNeuro repository (ds004504) [39] was selected. It includes EEG recordings from 88 subjects, of which 23 have frontotemporal dementia (FTD), 36 have Alzheimer’s disease (AD), and 29 are healthy control (HC) subjects. Participants were seated with eyes closed during the recordings (resting state) and were provided with the Mini-Mental State Examination test for cognitive and neurophysical assessment. Recordings were acquired at AHEPA University Hospital in Thessaloniki, Greece. The EEG2100 equipment from the Nihon Kohden Group was used. Nineteen scalp electrodes were used in line with the 10–20 international standard (Fp1, F7, F3, T3, C3, T5, P3, O1, Fz, Cz, Pz, Fp2, F4, F8, C4, T4, P4, T6, O2), with A1 and A2 used as references.

Recordings were sampled at 500 Hz, and the amplifier’s settings were tuned to 10 μV/mm, with a time constant of 0.3 s and a high-frequency filter of 70 Hz. Recording lengths varied between 13.5 min for the Alzheimer’s subjects and 12 min for the frontotemporal subjects, with 13.8 min for the healthy controls. Overall, the dataset had 485.5, 276.5, and 402 min of recordings, respectively, for AD, FTD, and healthy controls.

### 3.2. Pre-Processing Phase

The pre-processing phase associated with the EEG signals was initially executed by the researchers who recorded the data [39]. This included the execution of the Butterworth band-pass filter (0.5–45Hz), the re-referencing of channels to the A1 and A2 electrodes, and artefact elimination using ICA. The EEG data were cleaned from noise using Artefact Subspace Reconstruction, a method present in the EEGLab’s Matlab software. The RunICA algorithm was run to transform the nineteen EEG channels into independent components. Those containing *ocular* noise or *jaw* artefacts, via visual inspection, were zeroed, and inverse Independent Component Analysis (ICA) was executed. We extended preprocessing by using a sliding window technique to segment EEG data into overlapping 1-second windows, applying two strategies of 50% and 90% overlap, consistent with methods used in similar studies [40,41,42,43]. The rationale for contrasting two distinct overlapping window strategies was to evaluate the capability to extract time-domain biomarkers from limited EEG data and, thus, the feature extraction’s efficacy in discerning AD and FTD’s key attributes. Note that a 50% overlap strategy is faster than a 90% overlap strategy. Each window containing 500 data points (because of the 500 Hz sampling rate) led to the separate execution of the various selected feature extraction techniques and their different outputs. Each technique yielded 19 columns, the EEG channels, and *N* rows, the segmented overlapped EEG windows, for each subject. Subsequently, a concatenation of these individual tables was performed, leading to a final data shape of *N* (total windows) × window length (in seconds) × sampling rate (500 Hz) × 36 (number of subjects) columns and 19 rows (number of channels). The dataset was unbalanced across the target feature (class of subjects, AD/FTD, HC); thus, the Synthetic Minority Oversampling Technique (SMOTE) was applied. In detail, oversampling was performed for the 23 subjects with frontotemporal dementia (FTD) and the 29 healthy controls (HC) to match the 36 subjects with Alzheimer’s disease (AD).

### 3.3. Feature Extraction Techniques

As mentioned above, several techniques for feature extraction from EEG data were considered. These include the Singular Value Decomposition (SVD) Entropy, the Higuchi Dimension (HFD) based on fractal geometry, the Zero-Crossing Rate statistical indicator, the Detrended Fluctuation Analysis (DFA), and the Hjorth indicator. The PyEEG and Antropy Python libraries were used, and each individual technique is concisely detailed in the following parts of the text.

The Singular Value Decomposition (SVD) Entropy indicator is based on time series complexity. It evaluates the necessary number of orthogonal vectors for accurate data representation [44,45,46]. Mathematically,

(1) $$H_{SVD}=-\sum_{i=1}^{M}{\bar{\sigma }}_{i}\log_{2}\left({\bar{\sigma }}_{i}\right)$$

SVD Entropy correlates with the complexity of the underlying data, where ${\bar{\sigma }}_{i}$ is the embedded matrix’s normalised singular values.

The Higuchi Fractal Dimension (HFD) technique focuses on the non-parametric time series analysis based on generating new synthetic signals by a systematic procedure that sub-samples from the original data [47]. Mathematically,

(2) $$X^{mk}=\;X^{\left(m\right)},\;X^{\left(m+k\right)},\;X^{\left(m+2k\right)},\;\ldots \ldots ,\;X^{\left(m+\left\lfloor \frac{N-m}{k}\right\rfloor k\right)}$$

where *k* is the interval length, and *m* is the initial point. The time series is subsequently utilised to calculate the average curve length $L_{m}\left(k\right)$,

(3) $$L_{m}\left(k\right)=\frac{\sum_{i=1}^{\left\lfloor \frac{N-m}{k}\right\rfloor }|X^{\left(m\;+\;ik\right)}-X^{\left(m\;+\;(i-1)k\right)}\;|y}{k}$$

where term *y* is normalised and denoted as

(4) $$y=\frac{\left(N-1\right)}{\lfloor \frac{N-m}{k}\rfloor k}$$

$L_{m}\left(k\right)$ adheres to a power law, defining the Fractal Dimension *D*. HFD’s applicability to non-stationary series differentiates it from methods like Spectral and Hurst Exponents [48].

The Zero-Crossing Rate (ZCR) represents the rate of the change in the sign of a signal, essentially counting how many transitions of the zero amplitude exist within a time frame [49,50]. Mathematically,

(5) $$Z=\frac{\sum_{t=1}^{T-1}1_{R_{<0}}\left(s_{t}s_{t-1}\right)}{T-1}$$

where

- *Z* represents the Zero Crossing Rate.
- *T* is the total number of samples in the signal (or in a specified window/frame of the signal for localised analysis).
- $s_{t}$ and $s_{t-1}$ are consecutive samples in the signal at times *t* and t−1, respectively.
- $1_{R_{<0}}\left(s_{t}s_{t-1}\right)$ is an indicator function that evaluates to 1 if the product of $s_{t}$ and $s_{t-1}$ is less than 0 (indicating a zero crossing, where the sign of the signal changes between two consecutive samples), and 0 otherwise.
- The denominator (T−1) normalises the sum to account for the number of intervals between samples, providing a rate per sample interval.

The calculation starts by initialising a sum that will accumulate the total number of zero crossings. For each pair of consecutive samples $(s_{t}$ and $s_{t-1})$, starting from the second sample up to the last one, the product of these two samples is checked. The indicator function $1_{R_{<0}}\left(s_{t}s_{t-1}\right)$ checks if the product of $s_{t}$ and $s_{t-1}$ is negative. This is a mathematical way of determining if the sign of the signal changes between these two samples:

- If $s_{t}$ and $s_{t-1}$ have different signs, their product will be negative, indicating a zero crossing. The indicator function then contributes 1 to the sum.
- If $s_{t}$ and $s_{t-1}$ have the same sign, their product will be positive (or zero if either sample is zero, depending on how zero values are treated), and the indicator function contributes 0 to the sum.

The sum of all instances where the indicator function equals 1 gives the total number of zero crossings. This sum is then normalised by dividing by (T−1), which is the total number of intervals between consecutive samples in the signal or the window under consideration. The result of this division is the Zero Crossing Rate, *Z*, which gives a normalised measure of how frequently the signal crosses zero, thus providing insight into the signal’s properties, especially its frequency content and texture.

The Detrended Fluctuation Analysis (DFA) focuses on non-stationary time series for persistent patterns and correlations. It involves integrating the series, segmenting, detrending each segment, and calculating the fluctuation magnitude by contrasting the gradients of log(F(n)) and log(n).

(6) $$F\left(n\right)=\sqrt{\frac{\sum_{k=1}^{N}{\left[y\left(k\right)\;-\;y^{n}\left(k\right)\right]}^{2}}{n}}$$

where the detrending step is $y\left(k\right)-y_{n}\left(k\right)$.

The Hjorth parameters can be used to gauge insights into the characteristics of a signal, such as its regularity and frequency [46]. Three main parameters exist, namely, Activity, Mobility, and Complexity. Mathematically:

(7) Activity=var(y(t))

(8) $$Mobility=\sqrt{\frac{var\left(\frac{dy(t)}{dt}\right)}{var(y(t))}}$$

(9) $$Complexity=\frac{Mobility\left(\frac{dy(t)}{dt}\right)}{Mobility(y(t))}$$

Activity measures signal variance, mobility indicates signal frequency, and complexity assesses frequency variation. This research employs the average values of complexity and mobility derived from EEG signals.

### 3.4. Classification

The aforementioned techniques lead to features that are the input of various machine learning algorithms. A preliminary investigation of many learning algorithms was performed with the features extracted using the SVD Entropy technique and a 50% overlapping strategy among EEG windows. A 15-fold cross-validation strategy was employed, given the limited number of participants in the selected datasets. This preliminary training aimed to deliver an initial understanding of the capacity of each learning algorithm to fit the target feature (AD, FTD, HC) and minimise the computational power and time required for developing many models. The performance measures included the accuracy, precision, recall, F1-score averages, and area under the ROC curve (AUC) averages of the 15 surrogate models (Table 1).

We selected the four top-performing learning techniques based on their preliminary results (Table 1) to conduct a more focused comparative analysis, integrating sliding window techniques and feature extraction measures. This selection aimed to enhance the efficiency and depth of our model evaluation process by concentrating on the most promising algorithms. The four models are described in detail as follows:

1. K-Nearest Neighbors (KNN)—This technique can be used for supervised classification and regression, assuming that similar instances of a dataset cluster together. It is non-parametric and uses proximity to make classifications about the clustering of a new data point.
2. Random Forest (RF)—This technique constructs numerous decision trees during training. On the one hand, a supervised classification determines the most frequent class predicted by the single individual trees. On the other hand, for regression problems, it computes the average of such predictions. It incorporates randomness and includes sampling data with a replacement step to prevent model overfitting for individual tree learning by focusing only on a sub-set of data at each iterative tree’s split.
3. XGBoost—It is an enhanced and efficient form of Gradient Boosting that integrates regularisation as a form of model complexity control to mitigate overfitting. It also includes system-level enhancements to improve efficiency and flexibility, forming a robust predictive ensemble learning technique.
4. Extra Trees (ET)—It is an ensemble of decision trees with additional randomness. It not only bootstraps data but also chooses random split points for features. This extra randomness may reduce variance and improve the generalisation of new data.

The above data-driven learning techniques were subsequently trained on the features extracted using the aforementioned techniques (Table 1). In these circumstances, the dataset was partitioned into two by forming training and test sets (80 and 20% of the original data). To circumvent the risk of data leakage at the subject level, stringent actions were taken to ensure that the training and testing sets were devoid of features extracted from identical subjects. This was meticulously verified by maintaining subject-specific annotations throughout the entirety of the feature extraction process. For instance, consider a scenario where the training set includes subjects 1 to 4. This process ensured that the EEG features associated with these subjects were not in the testing set. Any duplicated features spotted in the testing set were iteratively moved into the training data. It aimed to guarantee the absence of subject data overlap between the sets while maintaining the 80:20 data distribution. Given the limited subjects, the training set was stratified using the 15-fold cross-validation. Through this approach, we divided the training data into 15 folds, using 14 for training and one for validation at each iteration. The top-performing models were selected and further tested on the unbalanced test set (20% of the overall data). Note that these test data were not augmented with SMOTE but left intact, which means following their original nature. The above training mechanism was performed twice: once with the 50% and once with the 90% overlapping strategy among the EEG segmented windows. Three classification tasks were devised: one for discriminating AD patients from healthy controls, one for discriminating FTD patients from healthy controls, and one for discriminating AD from FTD patients.

To further enhance the robustness of the designed multi-phase pipeline across the three classification tasks (Figure 1), the best-performing configuration (feature extraction technique/learning strategy) was repeated by employing five different seeds. This was aimed at generating different training and testing data five times. The averaged metrics from these five runs shaped the final results. Following the classification phase, we employed topographical brain mapping techniques to improve our predictive models’ interpretability. These maps were instrumental in pinpointing the cerebral regions most crucial for distinguishing between Alzheimer’s disease (AD) patients, frontotemporal dementia (FTD) patients, and healthy controls (HC). We selected the classification model with the highest accuracy and computed a feature importance array for each feature extraction technique, as detailed in Section 3.3. This array delineated the significance of features derived from each EEG channel in accurately predicting AD and FTD. Subsequently, we visualised these feature importance scores on topographic brain maps, illuminating the brain areas with elevated significance in the classification process.

The primary objective of this analytical step was to derive deeper insights into the specific brain regions that are most influential in the differentiation between AD patients, FTD patients, and healthy controls, thereby enhancing our understanding of the neurophysiological underpinnings of these disorders. This approach not only aids in validating the predictive models but also contributes to the broader field of neuroscientific research by identifying potential biomarkers and neuroanatomical correlates of these neurodegenerative diseases.

### 3.5. Hyperparameter Tuning

Optimising model hyperparameters was systematically conducted utilising the GridSearchCV module in Python. The aim was to identify the most effective parameter configurations for each machine learning model examined. Summarised below are the optimal settings discovered for each model:

1. K-Nearest Neighbors (KNN):
  (a) *leaf_size*: *30*, indicating the smallest number of points a node can hold.
  (b) *metric*: Utilised *euclidean* to measure point distances.
  (c) *n_neighbors*: Set to *6*, denoting the count of neighbours involved in decision-making.
  (d) *p*: Configured as *2*, corresponding to the Euclidean distance.
  (e) *weights*: Applied as *uniform*, assigning equal weight to all neighbours.
2. Random Forest (RF):
  (a) *criterion*: *gini*, as the split quality metric.
  (b) *n_estimators*: *120*, determining the forest’s tree quantity.
  (c) *max_depth*: *None*, allowing trees to grow unrestricted.
  (d) *min_samples_split*: *28*, minimal samples to split an internal node.
  (e) *min_samples_leaf*: *10*, the lowest number of samples for a tree’s leaf node.
3. XGBoost:
  (a) *eta (learning_rate)*: *0.1*, to control overfitting by moderating step size.
  (b) *n_estimators*: *280*, defining the count of boosting stages to perform.
  (c) *max_depth*: *8*, limiting the tree depth.
  (d) *colsample_bytree*: *1*, determining the fraction of features selected for tree construction.
  (e) *reg_alpha*: *0.05*, introducing L1 regularisation.
4. Extra Trees (ET):
  (a) *criterion*: *gini*, for evaluating splits.
  (b) *n_estimators*: *150*, the tree count developed.
  (c) *max_depth*: *None*, implying no depth limitation.
  (d) *min_samples_split*: *30*, the minimum required samples to split a node.
  (e) *min_samples_leaf*: *15*, the smallest count of samples a leaf node must have.

### 3.6. Model Evaluation

Each trained model was evaluated using different evaluation metrics:

- *Sensitivity* quantifies the capability of a model to correctly identify the two groups of subjects (having Alzheimer’s disease or healthy adults). It represents the rate of true positives (TP) over the actual positives. A higher sensitivity is synonymous with the robustness of a model in reducing mis-classifications of AD patients from healthy controls.
  (10) $$Sensitivity=\left(\frac{TP}{TP+FN}\right)$$
- *Precision* reflects the accuracy of a model in predicting positive outcomes, calculated as the fraction of true positives amongst all instances classified as positive by the model. A high precision implies a reduction in the occurrence of false negatives.
  (11) $$Precision=\left(\frac{TP}{TP+FP}\right)$$
- *Accuracy* measures the overall correctness of a model’s classifications, expressed as the percentage of true overall predictions.
  (12) $$Accuracy=\left(\frac{TP+TN}{TP+TN+FP+FN}\right)$$
- *Area under the ROC curve* represents a model’s ability to discriminate between AD patients and HC. It depicts the true positive rate against the false positive rate (on the y and x axes, respectively) at varying thresholds. ROC stands for Receiver Operating Characteristic and is expressed mathematically as follows:
  (13) $$TPR=\left(\frac{TP}{T+FN}\right)$$
  (14) $$FPR=\left(\frac{FP}{FP+TN}\right)$$
  The AUC-ROC is an aggregate measure of the overall performance of a model, which is its capability to distinguish between positive and negative instances across all possible classification thresholds.
- *F1 score*: The F1 score is a measure used to evaluate the performance of a model or a test, especially in cases where the balance between precision and recall is crucial. It is essentially a way to capture the balance between the importance of precision—how many of the items identified were relevant—and recall—how many of the relevant items were identified. This score is particularly valuable in situations where an uneven class distribution exists. The F1 score is the harmonic mean of precision and recall, providing a single metric that balances both concerns. The formula to calculate it is as follows:
  (15) $$Accuracy=\left(2*\frac{Precision*Recall}{Precision+Recall}\right)$$

## 4. Results and Discussion

Table 2 shows the performance metrics (from Section 3.6) of our models, evaluated using the original, unbalanced dataset. On the one hand, the results demonstrate that Alzheimer’s disease subjects could be discriminated with a superior precision (94–96%) compared to the frontotemporal disease subjects and healthy controls. On the other hand, the discrimination of FTD subjects always had the lowest precision across comparisons (86–88%). Sensitivity scores were always above 90% across the three comparisons, along with the F1-scores.

The observed lower precision in discriminating frontotemporal dementia (FTD) subjects from healthy controls, as compared to discriminating Alzheimer’s disease (AD) subjects, can be attributed to several factors intrinsic to the nature of these neurological conditions and the characteristics of the EEG signals they produce. The discrepancy in outcomes using feature extraction techniques (SVD Entropy, Detrended Fluctuation Analysis, Zero Crossing Rate, Higuchi Fractal Dimensions, Hjorth parameters) and machine learning algorithms (XGBoost, Random Forest, Extra Trees) may stem from the following:

- Overlap in EEG signal characteristics: FTD and healthy control EEG signals might share more similar characteristics than those observed between AD and healthy controls. FTD, particularly in its early stages, can manifest subtle EEG changes that are less distinct than those seen in AD, where more pronounced disruptions in brain activity patterns are common. This overlap makes it challenging for the applied feature extraction techniques to capture distinctive features that accurately differentiate FTD from healthy brain activity.
- Sensitivity and specificity of features: The feature extraction techniques employed may have differing sensitivities and specificities to the pathological changes in brain activity characteristic of FTD versus AD. For instance, features that are highly sensitive to global cognitive decline and widespread neural network disruption in AD may not be as effective in detecting the more localised or less severe disruptions typical of FTD.
- Stage of the disease: The stage of disease at the time of EEG recording could also impact the precision of discrimination. Early-stage FTD may produce very subtle EEG abnormalities that are difficult to distinguish from normal ageing processes, whereas AD-related changes, such as increased slow-wave activity, might be more evident and easier to detect even at earlier stages.
- Technical and methodological limitations: The choice of window size for EEG analysis, preprocessing steps, and the specific parameters used in both feature extraction and machine learning algorithms could preferentially favour the detection of AD over FTD. Optimising these methodologies specifically for FTD might require adjustments to better capture the nuanced differences in EEG signals associated with FTD.
- Variability within FTD spectrum: FTD encompasses a spectrum of disorders with heterogeneous clinical presentations, including behavioural variant FTD (bvFTD) and primary progressive aphasias. This variability contributes to a wider range of EEG signal manifestations, complicating the task of identifying a consistent set of features that distinguish FTD patients from healthy individuals across all subtypes.

**Figure 2:**
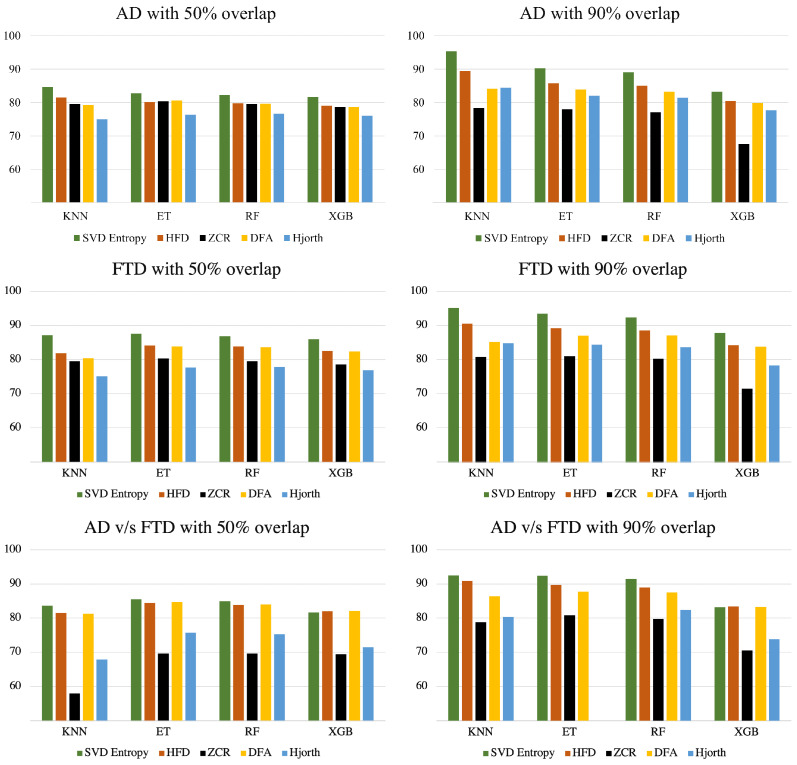
Average accuracy scores (over five runs) grouped by feature extraction technique and machine learning technique for Alzheimer’s/healthy controls (**top** row), frontotemporal/healthy controls (**middle** row), and Alzheimer’s/frontotemporal (**bottom** row) with the 50% overlap EEG windows strategy (**left**) and the 90% strategy (**right**).

Figure 2 shows the performance of models trained on balanced data with SMOTE in distinguishing Alzheimer’s disease subjects from healthy controls (top row), frontotemporal dementia subjects from healthy controls (middle row), and Alzheimer’s disease versus frontotemporal dementia subjects (bottom row) with the 50% and 90% overlapping windows strategy, over 5 runs. The details of these results are presented in Table 3, Table 4, Table 5, Table 6, Table 7 and Table 8.

**Figure 3:**
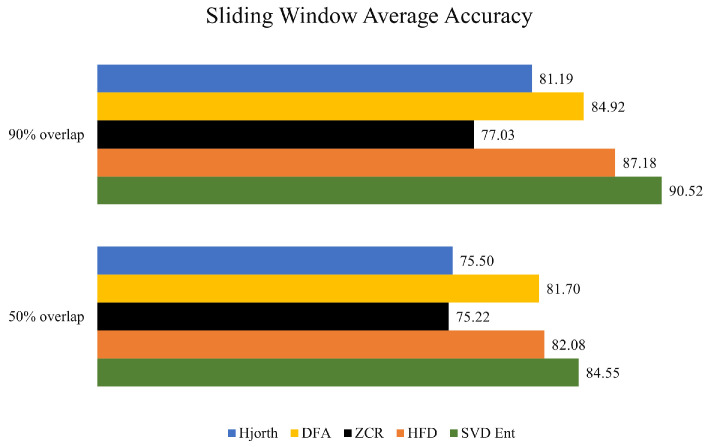
Average accuracies associated with the sliding window segmentation strategies of all the learning techniques grouped by feature extraction technique.

While consistent differences in accuracy across learning techniques have not been found, the Singular Value Decomposition Entropy technique seems to help to consistently develop predictive models with the highest accuracy, regardless of the underlying machine learning technique. This can also be observed from Figure 3 where the average accuracy of each sliding window is presented, grouped across the feature extraction measures. The same thing cannot be said for the other feature extraction techniques, which demonstrated no consistency across machine learning models and data overlapping strategies.

The higher accuracy and F1-score associated with SVD Entropy in this study can be attributed to the following functionalities that it exhibits:

- The Singular Value Decomposition (SVD) process breaks down the EEG signal into matrices that ignore the noise and preserve the principal characteristics of the EEG signal.
- Furthermore, SVD reduces the dimensionality of the data, abstracting it into a form that retains essential information while discarding redundancy. This abstraction makes it easier for machine learning models to process and learn from the data, enhancing predictive performance.
- Additionally, assessing the entropy in the distribution of singular values obtained from SVD quantifies the randomness and complexity of the signal. This is crucial for EEG analysis, where the complexity of brain activity can provide insights into neurological conditions.

Concerning the overlapping strategies, using the 90% overlapping strategy across consecutive EEG windows clearly exhibited utility when discriminating subjects with neurodegenerative disorders (AD and FTD versus HC).

**Figure 4:**
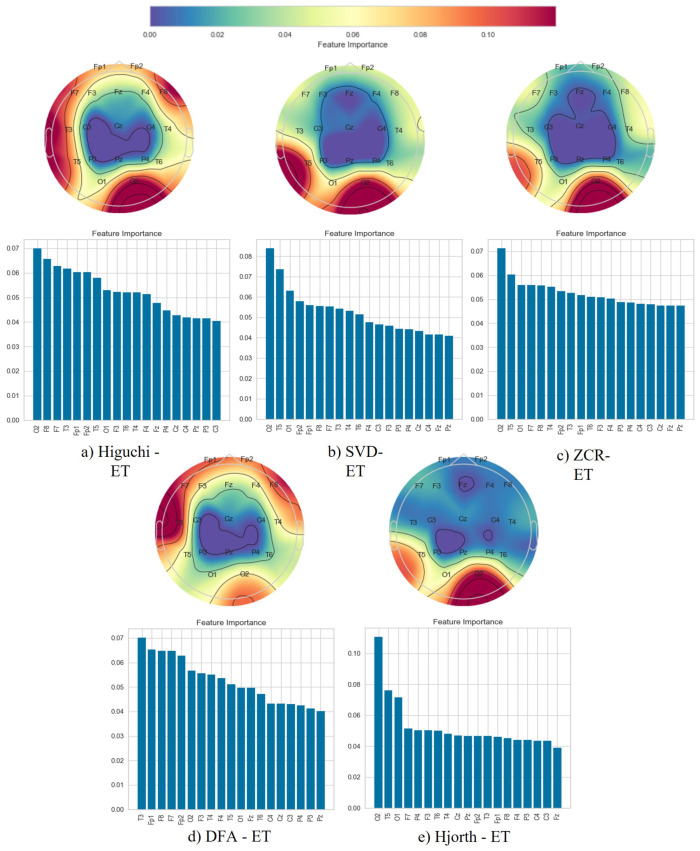
Feature importance map for discriminating Alzheimer’s disease subjects from healthy controls with the 50% window overlap.

**Figure 5:**
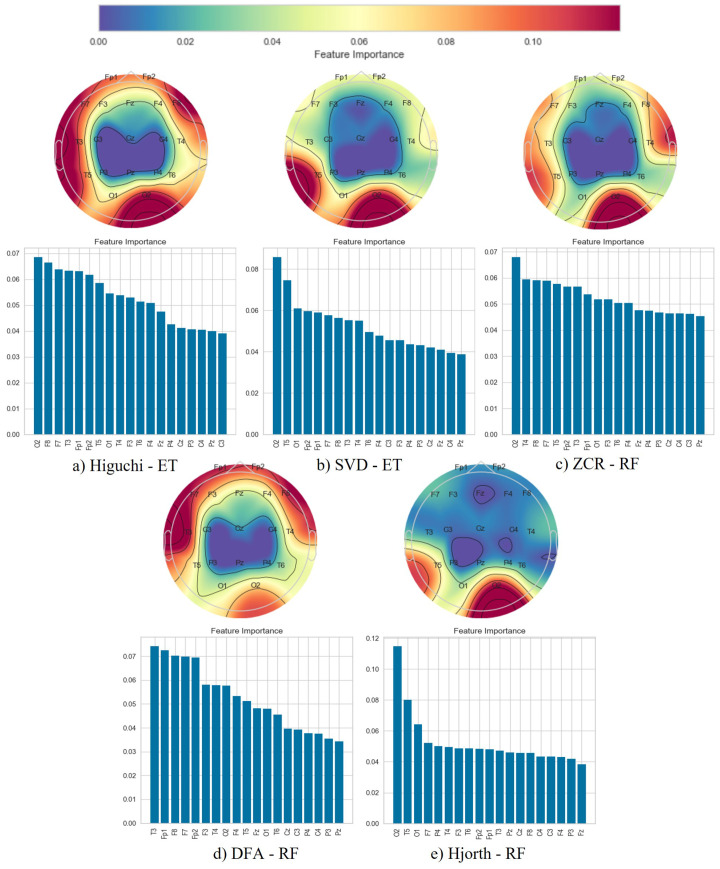
Feature importance map for discriminating Alzheimer’s disease subjects from healthy controls with the 90% window overlap.

**Figure 6:**
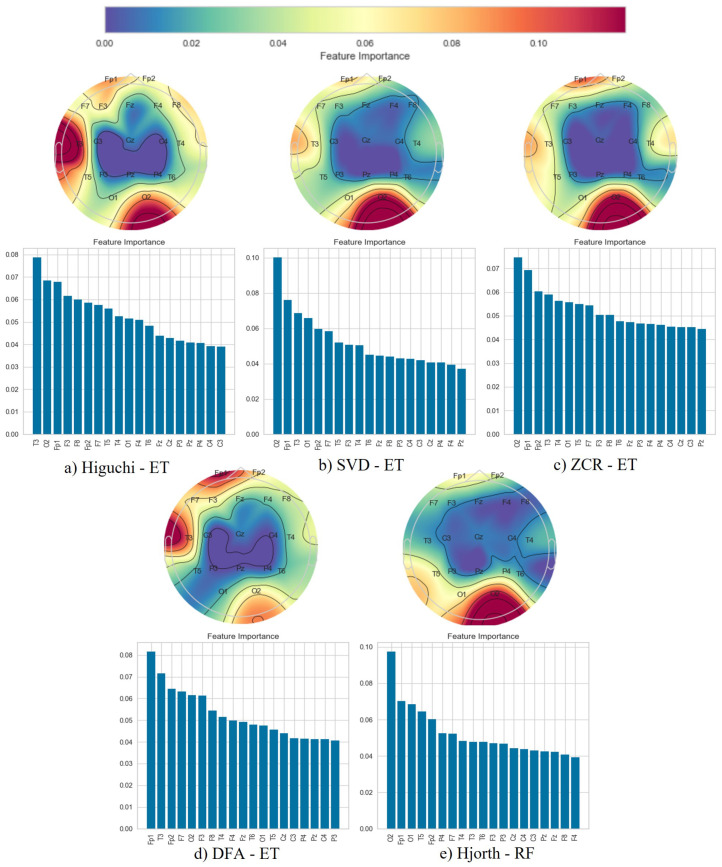
Feature importance map for discriminating frontotemporal dementia subjects from healthy controls with the 50% window overlap.

**Figure 7:**
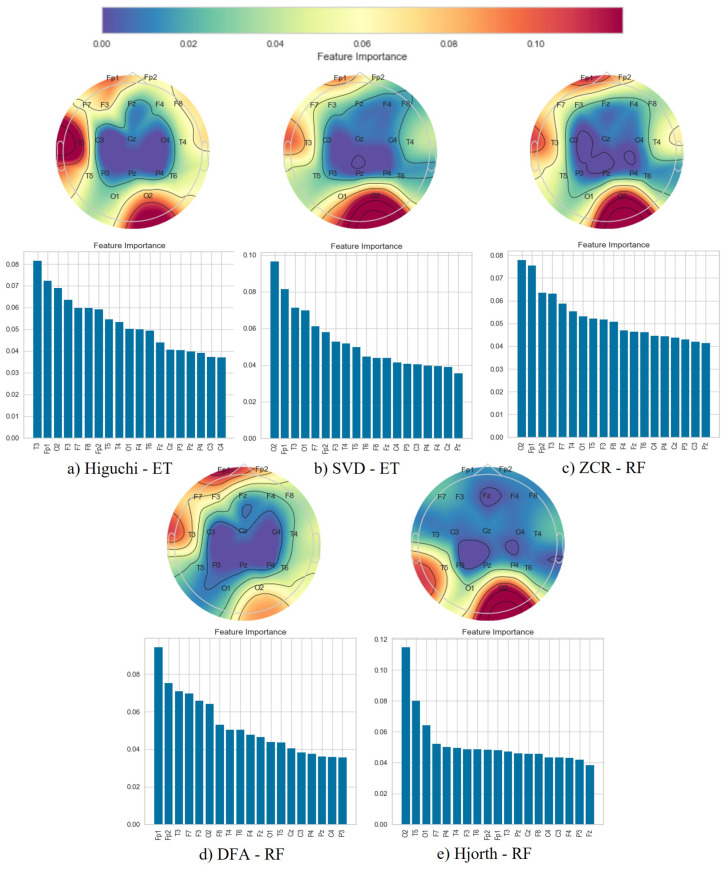
Feature importance map for discriminating frontotemporal dementia subjects from healthy controls with the 90% window overlap.

**Figure 8:**
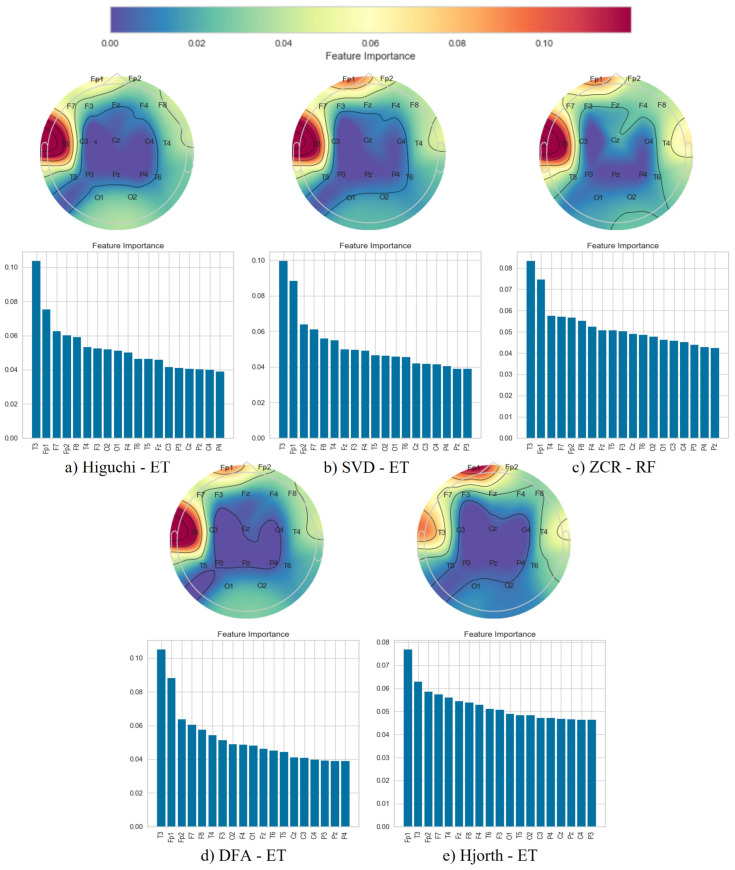
Feature importance map for discriminating Alzheimer’s disease subjects from frontotemporal dementia subjects with the 50% window overlap.

**Figure 9:**
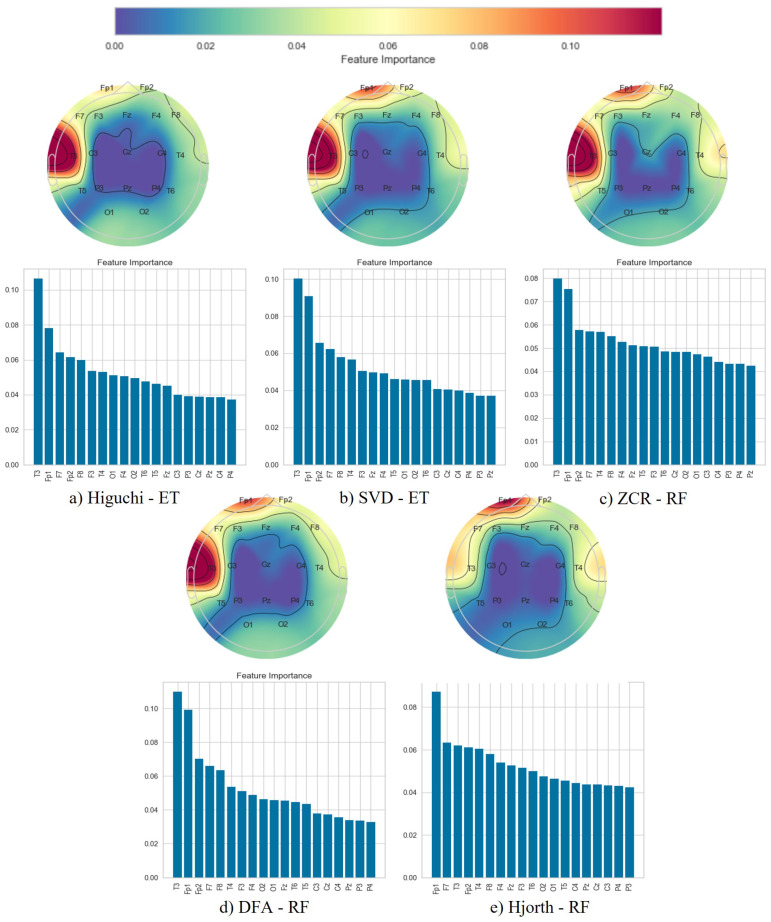
Feature importance map for discriminating Alzheimer’s disease subjects from frontotemporal dementia subjects with the 90% window overlap.

Following the classification phase, we employed a topographical brain mapping technique to improve the interpretability of our predictive models. These maps were instrumental in pinpointing the cerebral regions most crucial for distinguishing AD/FTD patients from HC and AD patients from FTD patients (Figure 4, Figure 5, Figure 6, Figure 7, Figure 8 and Figure 9). We selected the classification model with the highest accuracy (90% overlap, SVD entropy, and a tree classifier) and computed a feature importance array for each feature extraction technique, as detailed in Section 3.3. This array delineated the significance of features derived from each EEG channel in accurately predicting disease. Subsequently, we visualised these feature importance scores on topographic brain maps, illuminating the brain areas with elevated significance in the classification process. The primary objective of this analytical step was to derive deeper insights into the specific brain regions that are most influential in the differentiation between AD/FTD patients and healthy controls, thereby enhancing our understanding of the neurophysiological underpinnings of Alzheimer’s disease and frontotemporal dementia. This approach not only aids in validating the predictive models but also contributes to the broader field of neuroscientific research by identifying potential biomarkers and neuroanatomical correlates of these neurodegenerative diseases.

Topographic maps indicated the importance of the occipital, frontal, and temporal lobes in distinguishing AD from HC (Figure 4 and Figure 5), highlighting specific EEG channels (O2, T5, O1, Fp2, Fp1, F7, F8, T3, T4). Similar regions were critical for differentiating FTD from HC (Figure 6 and Figure 7), but with a different order of significance (O2, Fp1, T3, O1, F7, Fp2, F3, T4, T5), with a notable emphasis on the frontal lobe suggesting its effectiveness in capturing FTD features from the frontal region. In contrast to this, when differentiating AD patients from FTD patients (Figure 8 and Figure 9), the topographic plots show the frontal and temporal regions, especially channels T3, Fp1, Fp2, F7, F8, T4, F3, Fz, and F4, as being more important than the occipital region. This finding highlights a key distinction from feature importance patterns in AD/FTD vs. HC comparisons, where occipital dominance was observed.

Topographic analyses show the occipital, temporal, and frontal regions’ involvement in distinguishing AD from HC. This aligns with empirical observations about AD’s impact areas. The frontal and temporal regions are primarily involved in differentiating AD from FTD, which is consistent with FTD’s primary impact areas.

While topographic maps highlight the occipital region in distinguishing FTD from HC, this may seem unexpected given FTD’s primary impact on frontal and temporal regions [51,52]. However, this aligns with the involvement of the occipital lobe in advanced FTD stages, where it shares degeneration patterns with AD [53,54]. This overlap and variability in the FTD presentation underscores the need for accurate differential diagnosis between these diseases [54].

## 5. Conclusions

Alzheimer’s disease and frontotemporal dementia, resulting from neuronal damage, impair cognitive functions. Effective denoising and feature extraction from complex, noisy EEG data are essential for their early detection, focusing on dimensionality reduction and key biomarker identification.

Previous research on Alzheimer’s and frontotemporal dementia used limited feature extraction methods without thorough comparison. This study addressed this by evaluating multiple techniques for distinguishing AD and FTD conditions and healthy controls using EEG data. We trained models on features from EEG windows with 50% and 90% overlap, employing classifiers like K-Nearest Neighbors, Random Forest, XGBoost, and Extra Trees. The findings reveal that an increased overlap in EEG windows enhances model accuracy, particularly highlighting SVD entropy’s effectiveness over other techniques. Our model accurately distinguishes AD from FTD, pinpointing critical features in frontal, temporal, and occipital regions. This advances early-stage diagnosis by highlighting distinct EEG patterns specific to each disease.

Future directions include expanding this pipeline’s validation across broader datasets and more diverse subject groups, including AD, FTD, and healthy controls, and extending its utility to diagnose other neurodegenerative diseases like Schizophrenia and Parkinson’s disease. A further investigation is needed to determine the optimal EEG window overlap for effective feature extraction.

## Figures and Tables

**Table 1:** Average performance scores associated with the models trained with a 15-fold cross-validation, with a 50% overlap strategy among EEG windows, using the SVD Entropy feature extraction technique.

Learning Tech.	Accuracy	Precision	Sensitivity	F1-Score	AUC
KNN	84.70%	81.37%	85.90%	83.58%	91.82%
ET	82.72%	81.93%	79.43%	80.66%	91.06%
RF	82.22%	81.33%	78.94%	80.11%	90.42%
XGBoost	81.66%	78.87%	81.41%	80.12%	89.96%
LGBM	78.04%	75.08%	77.25%	76.15%	86.78%
GBC	73.38%	69.58%	73.42%	71.45%	81.09%
LDA	71.97%	68.99%	69.44%	69.21%	78.42%
Ridge	71.86%	68.81%	69.48%	69.14%	0.00%
DT	71.72%	68.41%	70.00%	69.20%	71.58%
LR	71.55%	68.23%	69.78%	69.00%	78.03%
SVM	69.81%	66.05%	69.90%	67.63%	0.00%
ADA	69.26%	65.40%	68.48%	66.90%	75.67%
QDA	66.79%	59.22%	86.10%	70.17%	79.84%
NB	57.12%	51.94%	73.67%	60.92%	64.33%

**Table 2:** Classification results associated with the model trained with the 90% overlap EEG window strategy on original (unbalanced) data using KNN classifier with SVD Entropy (Alzheimer’s disease subjects versus healthy controls in the top row, healthy controls versus frontotemporal dementia subjects in the middle row, and Alzheimer’s disease versus frontotemporal dementia subjects in bottom row).

Group	Precision	Sensitivity	F1-Score	Number of Windows
Alzheimer	0.94	0.91	0.94	58,119
Healthy controls	0.89	0.92	0.92	48,239
**Accuracy**			0.91	106,358
Healthy controls	0.96	0.92	0.94	48,095
Frontotemporal dementia	0.88	0.94	0.91	33,200
**Accuracy**			0.93	81,295
Alzheimer	0.96	0.90	0.93	58,008
Frontotemporal dementia	0.86	0.95	0.90	33,322
**Accuracy**			0.91	91,330

**Table 3:** Classification of Alzheimer’s disease from healthy adults with the 50% overlap strategy across the feature-extraction techniques for EEG data and the learning techniques.

Feature Extraction	Learning Techn.	Accuracy	Precision	Sensitivity	AUC
SVD	KNN	84.68	81.37	85.93	91.82
	ET	82.72	81.93	79.43	91.06
	RF	82.22	81.33	78.94	90.42
	XGB	81.66	78.87	81.41	89.96
HFD	KNN	81.51	80.56	78.10	90.21
	ET	80.32	78.69	77.65	88.90
	RF	80.02	77.92	78.11	88.42
	XGB	79.06	77.39	76.08	87.84
ZCR	ET	80.37	77.85	79.09	88.89
	KNN	79.53	75.07	81.95	87.35
	RF	79.53	76.66	78.69	88.16
	XGB	78.63	74.00	81.29	87.51
DFA	ET	80.54	78.25	79.01	88.98
	KNN	79.25	74.8	81.74	87.25
	RF	79.63	76.87	78.74	88.11
	XGB	78.64	74.08	81.32	87.34
Hjorth	RF	76.67	76.22	70.84	84.29
	ET	76.36	76.38	69.54	84.19
	XGB	76.04	74.05	72.89	84.18
	KNN	74.98	71.98	73.67	81.90

**Table 4:** Classification of Alzheimer’s disease from healthy adults with the 90% overlap strategy across the feature-extraction techniques for EEG data and the learning techniques.

Feature Extraction	Learning Techn.	Accuracy	Precision	Sensitivity	AUC
SVD	KNN	94.72	94.66	93.42	96.64
	ET	90.24	89.72	88.59	96.64
	RF	89.10	88.41	87.38	95.81
	XGB	83.19	80.35	83.23	91.62
HFD	KNN	89.43	86.87	90.30	95.79
	ET	85.76	83.82	84.92	93.77
	RF	85.07	82.67	84.78	93.13
	XGB	80.45	76.26	82.47	89.47
ZCR	KNN	78.41	73.51	81.75	86.36
	ET	77.99	77.17	72.87	86.96
	XGB	67.57	65.09	60.99	74.21
	RF	77.10	76.59	71.08	85.74
DFA	KNN	84.07	80.41	85.68	91.61
	ET	83.91	81.41	83.51	92.20
	XGB	79.86	74.98	83.28	88.82
	RF	83.27	80.28	83.57	91.62
Hjorth	KNN	84.41	82.28	83.61	91.64
	ET	82.02	82.71	76.30	90.22
	RF	81.49	81.51	76.53	89.59
	XGB	77.68	75.90	74.41	86.01

**Table 5:** Classification of frontotemporal dementia from healthy adults with the 50% overlap strategy across the feature-extraction techniques for EEG data and the learning techniques.

Feature Extraction	Learning Techn.	Accuracy	Precision	Sensitivity	AUC
SVD	ET	87.57	85.52	83.61	94.74
	KNN	87.21	80.36	90.75	94.10
	RF	86.85	83.47	84.40	94.25
	XGB	86.01	81.49	84.90	93.70
HFD	ET	84.11	82.40	77.56	91.72
	RF	83.86	80.92	79.04	91.43
	XGB	82.53	76.63	82.21	91.18
	KNN	81.84	73.29	87.23	90.36
ZCR	ET	80.37	77.85	79.09	88.89
	KNN	79.53	75.07	81.95	87.35
	RF	79.53	76.66	78.69	88.16
	XGB	78.63	74.00	81.29	87.51
DFA	ET	83.87	85.84	72.58	91.16
	RF	83.62	83.76	74.43	90.99
	XGB	82.34	78.16	78.92	90.47
	KNN	80.45	72.18	85.05	89.37
Hjorth	RF	77.85	71.87	74.74	86.29
	ET	77.69	72.77	72.43	86.50
	KNN	75.10	65.64	81.82	83.83
	XGB	76.94	68.99	79.03	86.34

**Table 6:** Classification of frontotemporal dementia from healthy adults with the 90% overlap strategy across the feature-extraction techniques for EEG data and the learning techniques.

Feature Extraction	Learning Techn.	Accuracy	Precision	Sensitivity	AUC
SVD	KNN	94.18	90.49	95.85	98.23
	ET	93.48	92.98	90.88	98.29
	RF	92.41	90.99	90.35	97.68
	XGB	87.81	83.51	87.36	95.14
HFD	KNN	90.52	84.59	93.81	96.86
	ET	89.20	88.96	83.89	95.73
	RF	88.58	87.16	84.37	95.25
	XGB	84.24	78.58	84.28	92.65
ZCR	ET	80.96	83.63	66.15	89.16
	KNN	80.81	73.38	82.89	88.97
	RF	80.22	81.85	66.03	87.94
	XGB	71.44	69.35	53.38	77.47
DFA	RF	87.05	87.96	79.02	94.02
	ET	86.99	89.48	77.12	94.09
	KNN	85.17	77.71	89.17	93.17
	XGB	83.83	79.71	80.90	91.84
Hjorth	KNN	84.80	76.85	89.72	93.08
	ET	84.39	81.03	80.53	92.59
	RF	83.62	79.12	81.21	91.82
	XGB	78.28	70.03	81.59	88.21

**Table 7:** Classification of Alzheimer’s disease from frontotemporal dementia adult subjects with the 50% overlap strategy across the feature-extraction techniques for EEG data and the learning techniques.

Feature Extraction	Learning Techn.	Accuracy	Precision	Sensitivity	AUC
SVD	ET	85.50	85.45	92.43	94.28
	RF	84.96	82.15	74.88	91.82
	KNN	83.60	73.23	86.50	90.30
	XGB	81.58	73.15	77.94	89.21
HFD	ET	84.43	81.49	73.78	91.58
	RF	83.84	78.83	75.74	90.97
	XGB	81.97	72.62	80.62	90.2
	KNN	81.51	80.56	78.10	90.21
ZCR	RF	69.60	66.53	31.94	68.06
	ET	69.57	66.45	31.89	68.55
	XGB	69.38	66.75	30.45	68.86
	KNN	57.98	44.43	64.96	63.94
DFA	ET	84.71	82.78	73.11	91.68
	RF	84.02	79.75	75.05	91.17
	XGB	82.13	73.12	80.35	90.23
	KNN	81.25	69.19	87.21	90.27
Hjorth	ET	75.47	75.09	49.82	80.74
	RF	75.26	69.96	56.04	80.39
	XGB	71.48	59.95	65.04	78.52
	KNN	67.87	54.30	73.71	76.48

**Table 8:** Classification of Alzheimer’s disease from frontotemporal dementia adults with the 90% overlap strategy across the feature-extraction techniques for EEG data and the learning techniques.

Feature Extraction	Learning Techn.	Accuracy	Precision	Sensitivity	AUC
SVD	KNN	91.93	85.03	94.37	97.48
	ET	92.37	93.45	84.93	97.74
	RF	91.42	90.81	84.96	97.07
	XGB	83.17	74.80	80.83	91.15
HFD	KNN	90.88	88.24	82.90	97.11
	ET	89.67	88.51	82.16	95.95
	RF	88.93	86.09	82.85	95.32
	XGB	83.43	74.03	83.66	91.87
ZCR	RF	79.72	85.79	52.79	86.41
	KNN	78.81	66.53	83.61	87.98
	XGB	70.50	70.68	31.81	71.57
	ET	80.81	86.63	55.63	87.74
DFA	RF	87.52	84.81	79.96	94.30
	KNN	86.37	76.16	90.95	94.11
	ET	87.78	86.54	78.58	94.52
	XGB	83.23	74.14	82.66	91.55
Hjorth	RF	82.36	81.16	66.92	89.38
	ET	80.30	68.44	84.86	89.53
	XGB	73.79	63.03	67.23	81.48
	KNN	67.87	54.30	73.71	76.48

## Data Availability

The EEG data utilised in our study were sourced from the OpenNeuro repository, which exclusively hosts datasets that have received ethical approval for public sharing and use. The dataset documentation confirms ethical approvals in accordance with the Declaration of Helsinki and approval by the Scientific and Ethics Committee of AHEPA University Hospital, Aristotle University of Thessaloniki, under protocol number 142/12-04-2023. Data and ethical approvals can be viewed online at https://openneuro.org/datasets/ds004504/versions/1.0.7 (accessed on 15 March 2023).
